# The Pathology and Treatment of Leucorrhœa

**Published:** 1855-11

**Authors:** 


Art. IV.—*The Pathology and Treatment of Leucorrhœa.* By W. Tyler Smith, M. D., Member of the Royal College of Physicians; Physician Accoucheur to St. Mary's Hospital; Lecturer on Midwifery and the Diseases of Women in St. Mary's Hospital Medical School; Vice-President of the Medical Society of London; Honary Fellow of the Obstetrical Society of Dublin, etc., etc. Philadelphia: Blanchard & Lea. 1855. pp. 199. 8vo.

---

Every practitioner of medicine has been made keenly to feel how much the subject of uterine and vaginal disorders is in need of ampler investigation. Industriously as they have been studied, especially within a few years, there are still great difficulties attending their treatment, and it is therefore that we hail with pleasure this new contribution to one branch of the subject. Dr. Smith has studied the discharges from the vagina microscopically, and thinks he has derived from their appearance important aid in the diagnosis of uterine and vaginal disorders. He precedes his account of these discharges with a description of the vagina, os, and cervix uteri as they are seen under the microscope, which is illustrated with numerous and highly instructive wood cuts. The secretions of these parts in the healthy state are then described, after which he takes up the different forms of leucorrhœa. He remarks:—

"All pathology has its basis in physiology. In the various departments of medicine, the only way of reconciling differences of opinion is by investigation. The demonstration of two very differently organized surfaces in the vagina, and in the canal of the cervix uteri, with the existence of two very distinct forms of secretion, naturally lead us to the consideration of two principal forms of leucorrhœa. But at this point it may be well to revert for a moment to the special differences which exist between the vagina and the cervical canal. The lining membrane of the vagina approaches in organization to the skin; it is covered by a thick layer of scaly epithelium; it contains in the greater part of its surface few if any mucous follicles or glands; its secretion is acid, consisting entirely of plasma and epithelium, and the chief object of the secretion is the lubrication of the surface upon which it is formed. On the other hand, the lining of the canal of the cervix is a true mucous membrane; it is covered in great part by cylinder epithelium; it abounds with immense numbers of mucous follicles having a special arrangement; it pours forth a true mucous secretion, alkaline in character, and consisting of mucus-corpuscles and plasma, with little or no epithelium; and this secretion has special uses to perform in the unimpregnated state, and in pregnancy and parturition. Leucorrhœa admits of a similar division. The first and the most frequent and important is the Mucous variety, consisting chiefly of mucus-corpuscles and plasma, and secreted chiefly by the follicular canal of the cervix. The second is the Epithelial variety, in which the discharge is Vaginal, or is secreted by the vaginal portion of the os and cervix, and consists for the most part of scaly epithelium and *débris*. These two varieties may of course exist in various degrees of combination; sometimes the one and sometimes the other preponderates, or is the original affection; but the chief importance must be given to cervical or mucous leucorrhœa, as being the most obstinate and common. I pass by at present altogether those discharges limited to the ostium vaginæ, as these, when they occur, which is seldom, are easy of diagnosis and cure. I have already referred to the antithesis of the function which exists between the cervical canal, and the cavity of the fundus uteri; the one secreting mucus periodically, the other producing the catamenial secretion. It is impossible not to be struck with certain points of comparison and contrast between some of the disorders of the fundus uteri and cervical leucorrhœa. In certain cases of menorrhagia, for instance, the periodical sanguineous discharge is converted into a constant colored discharge; while in severe cases of leucorrhœa the periodical white mucous discharge is rendered permanent. I shall have hereafter to show that the relations between leucorrhœa and the catamenial function in health and disease are very interesting and important."

After describing these varieties of leucorrhœa, the author proceeds to point out the sequelae of the affection, which are inflammation, abrasion, ulceration, induration, and hypertrophy of the os and cervix uteri, and abrasion and superficial ulceration of the vagina. He then indicates certain relations found to exist, occasionally, between secondary syphilis and leucorrhœa, and in subsequent chapters the relations of leucorrhœa to disorders of the function of menstruation, and to sterility and abortion. The concluding chapters treat of the constitutional and local causes of leucorrhœa, and its treatment. Among the causes he enumerates plethora, debility, prolonged lactation, strumous habit, skin diseases, climate, rectal irritation, vesical and urethral irritation, vaginal and uterine irritation, gestation and abortion. We pass to what he says of the treatment:—

"In the treatment of leucorrhœa, as of all other uterine disorders, it must never be forgotten that the principle of growth, and the tendency to perverted nutrition, is more active in the female reproductive organs than in any other parts of the economy. This exuberance is seen not only in the profuse growth of tumors of various kinds, but in the active discharges, both menorrhagic and leucorrhœal, to which the uterus is liable, and in the changes by which they are accompanied. The only way in which the nutrition and secretions of these parts can be restrained within a proper limit, when there is a tendency to excess or want of balance, is by keeping the whole of the organs and, as far as possible, all their various functions, in a state of healthful but not excessive activity.

"In such cases the particular constitutional state found in combination with leucorrhœa should be met, as far as possible, by medicines, diet, and regimen, especial attention being in all cases shown to the disorders of menstruation. It will be found that very few cases are met with in which menstrual disorder of some kind is not a part and complication. Into the particular treatment of such cases I need not enter; it will probably be more useful if I make some remarks upon the various remedies in use, and the methods of treatment, both of constitutional and local character. In leucorrhœa having a specific origin, as in the cases referred to in chapter vi., of course a special treatment becomes necessary. No means avail in curing the lucorrhœal discharge, and the other uterine symptoms, without the removal of the constitutional taint of syphilis.

"*Preparations of Iron.*—No single remedy is of equal importance with steel, in the treatment of leucorrhœa attended or caused by debility. In patients of strumous habit, the iodide of iron is of great value. In cases attended by gastric irritability, the ammonio-citrate, or the citrate of iron; and in cases attended by relaxation of the system, the sesquichloride of iron, and the sulphate of iron, are very valuable. The various chalybeates of this country and the Continent are often of great use, particularly in cases where only small doses of iron can be borne by the stomach. Some persons affected with lucorrhœa require a suitable preparation of steel almost habitually, as part of their diet, rather than as medicine. Even in leucorrhœa occurring in plethoric habits, it is often necessary to give iron in combination with aperients and alteratives. The chief objection to the use of iron in feeble habits, occurs in menorrhagic cases, in which only astringent preparations of iron are admissible. Sometimes the sesquichloride, or the iron alum, is useful during the actual flow of the menses. In other cases these preparations increase the loss, if given during the periods, but are of great use during the intervals. In cases of leucorrhœa combined with amenorrhœa in feeble habits, and in periodical leucorrhœa, the preparations of steel are the only medicines which can be relied upon, the re-establishment of the catemenia being the surest method of removing the leucorrhœa.

"*Iron Alums.*—A combination of alum and iron has long been used in the treatment of leucorrhœal disorders. The efficacy of the Sand-rock chalybeate water, in the Isle of Wight, in these affections, is tolerably well known. This spring is powerfully impregnated with sulphate of iron and sulphate of alumina, upwards of forty-one grains of the former and thirty-one of the latter being contained in every pint. At the commencement of the present century, this water was extensively used by Dr. Saunders and by several physicians engaged in the treatment of uterine disease, having been introduced to the notice of the profession by Mr. Waterworth, of Newport, whose excellent accounts of the spring and its medicinal properties have been lately republished by his son. From the analysis of Dr. Marcet, it would appear that the sulphate of iron and alumina are not in chemical combination, but are simply dissolved together in the water. The springs of Hartfell, in Scotland, and Horley-green, in Yorkshire, and several springs on the Continent, have a similar composition, but are less powerful than the Sand-rock water. There can be no doubt of the curative effects of this water, and it is still resorted to in various diseases of relaxation and debility, and is exported in considerable quantities to India and other warm climates. My friend, Mr. J. R. Martin, has always used it extensively in the disorders of females on their return from tropical climates, particularly in cases of leucorrhœa and menorrhagia.

"In imitation of this mineral water I was in the habit of prescribing for my hospital patients a mixture of sulphate of iron and common alum in water, in the proportion of from two to five grs. of each, dissolved in water, two or three times a day, with very good effect, when I learned from Mr. Lindsey Blyth, the late disspenser at St. Mary's Hospital, who is a most excellent pharmaceutical chemist, that Mr. Davenport had placed some beautiful crystals of a preparation of iron alum in the Great Exhibition in the year 1851. This preparation was obtained in a casual manner by Mr. Davenport from a solution of persulphate of iron. At my request Mr. Blyth procured some of this salt, and in 1852 I began to administer it in cases of leucorrhœa, prior to which I believe it had never been used in medicine. I found it remarkably efficacious, and have constantly prescribed it since that time. I certainly do not know of any other internal remedy which at all equals it in leucorrhœa. Since I began to use it, it has been employed by my colleagues, and by other physicians, but sometimes other compounds have been used under the name of iron alum. Dr. Bence Jones, for instance, in a lecture in the Medical Times, (Nov. 12, 1853), stated that he had used iron alum with good effect, having obtained it from Mr. Morson, of Southampton Row. From an inquiry of Mr. Morson, I found the preparation he had supplied was merely a mixture of sulphate of iron and sulphate of alumina and potash, and not the true iron alum."

After mentioning tonics, and purgatives, and injections, and stating the circumstances under which they are to be employed, he proceeds to speak of caustic applications, respecting which he says:—

"In this place I wish to say a few words on the use of violent caustics in the treatment of uterine disease, and particularly of the so-called ulceration and hypertrophy of the os and cervix uteri. One point appears to me to have been almost lost sight of in the use of these applications. The uterus has to pass physiologically through great changes of growth and development in the course of pregnancy, parturition, and the return of the organ to the unimpregnated state. Of all the changes which occur to the uterus during the evolution and devolution of the organ before and after delivery, those which occur in the os and cervix uteri are perhaps the most remarkable. In the great majority of patients who present themselves for treatment, it must not be forgotten, that the uterus will have at some future time to pass through these changes. This circumstance alone appears to me to furnish a very strong *à priori* argument against the use of caustics sufficiently strong to cause disorganization of the os and cervix uteri. My own opinion is that the potassa fusa, the potassa cum calce, the acid nitrate of mercury, the chloride of zinc, and the actual cautery, are on this account rarely, if ever, admissible in the treatment of non-malignant disease of the uterus. It has been said that a hypertrophied cervix can be melted down by the use of these destructive agents; but this simply means that portions of the os and cervix uteri may, like other soft tissues, be destroyed by caustic; for it cannot be contended that when violent escharotics are applied to the uterus, the morbid elements are alone affected, the proper structure of the organ remaining intact. The evils of such applications are, as it appears to me, some of them immediate; others are remote. It is the fact that in the use of these violent caustics the death of the patient has been caused by perforation of the vagina behind the posterior lip of the uterus, and the occurrence of fatal peritonitis. Pelvic inflammation, ending in abscess, is another unfortunate result of this practice. I have also known death to occur from cutting off portions of a hypertrophied uterine lip with the knife. No doubt some practitioners apply the more heroic caustics with such care as to avoid any immediate risk; but I doubt if it be possible, with the utmost caution, to prevent ultimate mischief. In Case X., p. 75, I have related the instance of a lady treated by a very skillful practitioner, in whom the melting down of the os and cervix uteri was followed by extensive giving way of the cicatrices, upon the occurrence of pregnancy. I have seen other cases of a similar kind. I have also met with cases in which the firmest adhesions have formed between the os uteri and the vagina, after deep cauterizations. Three years ago a patient came to me who had apparently suffered from leucorrhœa and hypertrophy of the cervix uteri for many years, and who at length, under the care of a practitioner of large experience in a distant colony, had had a prolonged anterior uterine lip excised, and the cervix uteri treated liberally with potassa fusa and potassa cum calce. This patient was seen separately by Dr. Locock and myself, and it was found that the os uteri had, in cicatrizing, completely adhered to the upper part of the posterior vaginal wall. The case was sent to England as one of carcinoma uteri; but the appearances which were considered to indicate cancer really arose from hypertrophy, and the irregular cicatrizations consequent upon excessive cauterization. In this case the shape of the os and cervix uteri was entirely lost. The subject of it will probably never become pregnant; she has not done so up to the present time; but if she should conceive, the os uteri will have to be torn from the rectum. The upper part of the vagina is in much the same state as we sometimes find it after instrumental labors, in which sloughing and cicatrization have taken place. In other cases the os uteri has been completely closed by adhesive inflammation, after the use of potassa fusa, rendering it necessary to puncture the os to relieve the uterus of retained menstrual fluid. One patient, suffering severely from long-standing scrofulous disease of the elbow and knee joints, and bearing the most extensive marks of scrofulous ulceration, came to me at St. Mary's Hospital, from the country, in whom the whole of the lower part of the cervix uteri had been removed by the frequent application of caustic potash, though the leucorrhœa had evidently depended on the constitutional disorder. I have at the present time an in-patient, in whom the whole of the lower part of the cervix was formerly destroyed by potassa fusa, but she still suffered from profuse leucorrhœa on her admission.

"Such are some of the evils which follow the use of the more violent escharotics in the treatment of ordinary uterine disease. It may be asked, are there no positive advantages in their use, which outweigh the disadvantages of such accidents? In my opinion there is no good which can be effected by the more powerful caustics, which cannot be accomplished by the nitrate of silver, or by other means. It is true that by the prolonged application of the nitrate of silver, loss of substance may be caused, but this is far less likely to occur with lunar caustic than with the more powerful escharotics. It is also true that some practitioners apply the more violent caustics so lightly that they do not exceed the milder medical action of the solid nitrate of silver, but in such cases it would be quite as well to use the safer remedy where a caustic is required."

We believe this work will prove a valuable addition to our literature on the interesting subject of which it treats, and will supply the practitioner with the means of making a more satisfactory diagnosis in this class of affections. We need hardly add, that the publishers have reproduced it in the best style of their art.

